# Experiences and wishes of women regarding systemic aspects of midwifery care in Germany: a qualitative study with focus groups

**DOI:** 10.1186/s12884-017-1552-9

**Published:** 2017-11-21

**Authors:** Elke Mattern, Susanne Lohmann, Gertrud M. Ayerle

**Affiliations:** Martin-Luther-Universität Halle-Wittenberg, Medizinische Fakultät, Institut für Gesundheits- und Pflegewissenschaft, Magdeburger Straße 8, 06112 Halle (Saale), Germany

**Keywords:** Preferences, Deficits, Pregnant women, Mothers, Midwifery care, Exploratory study, Focus groups, Germany

## Abstract

**Background:**

Knowledge of pregnant women’s and mothers’ viewpoints on midwifery care is crucial for its appropriate delivery and research. In Germany, comprehensive research to more fully understand women’s needs in pregnancy, labour, birth and the postpartum period until weaning is lacking. International studies provide some knowledge of women’s expectations, their choices, and subjective criteria indicating good midwifery care.

**Methods:**

This study explores pregnant women’s and mothers’ experiences, needs and wishes regarding systemic aspects of midwifery care (access, availability, choices, model of midwifery care; maternity care in the healthcare system). 50 women participated in 10 focus groups in 5 states of Germany. The groups were heterogeneous with regard to age, parity, model of maternity care used, and rating of satisfaction. Women with limited educational years (*n* = 9) were personally contacted by midwives and reached by social media. Also, mothers living in a mother-child home (*n* = 6) or attending a peer group for grieving parents (*n* = 5) were included. The digitally documented focus groups were systematically analysed in an itinerary hermeneutic manner.

**Results:**

Three themes were identified: (a) Knowledge or lack of awareness of midwifery care, (b) availability of and access to midwives, and (c) midwifery care in the healthcare system. Theme (a) entails the scope of midwifery care and the midwife’s competence, but also a lack of information, inconsistent counselling, and difficulty identifying midwives. Theme (b) encompasses aspects such as the availability, accessibility and selection of a midwife, the effort involved in looking for a midwife, the challenge of transition points, and family midwives. Theme (c) relates interprofessional cooperation, gaps/inadequacies of care during latency phase, alternative models of care, and the importance of family and peer groups for women.

**Conclusions:**

Midwifery care and research in Germany must address the issue of imparting relevant information about midwifery services. Interprofessional cooperation and management of transition points ought to be improved in the interests of the women concerned. Moreover, the quality of antenatal classes, support during latency phase, and intrapartum care in hospitals need to be addressed. Lastly, the special needs of vulnerable women in midwifery care must become a major focus in Germany.

## Background

Chalmers and Glasziou [1] und Chalmers et al. [2] have called for users’ input and even active participation in determining research topics and questions, so that researchers won’t study problems of health care, or outcomes, which are not vital to the respective population.

Internationally, some research projects have focussed on women’s experiences, needs and wishes. For example, Iravani et al. [3] explored women’s needs and expectations during normal labour and delivery in general, whereas other research more specifically addressed complex needs of vulnerable women [4] and maternity care needs in rural areas [5]. In Germany, there has been some research on the subjective views and experiences of women within the realm of midwifery care since the turn of the millennium [6–12]. However, comprehensive research within the context of the German healthcare system to better understand and appraise the interplay between women’s needs pertaining to and services rendered in midwifery care is lacking.

In the following article, contextual information on midwifery in Germany is given to assist in understanding the study results, also in terms of their broader significance for other countries with different healthcare systems. Secondly, the state of research in Europe regarding the experiences of women in midwifery care is presented, providing a background for the new knowledge generated by the study.

### Midwifery care within the German healthcare system

In Germany, an estimated 21,000 midwives care for about 700,000 childbearing women, annually. By law [13], every woman in Germany is entitled to midwifery care from conception until introduction of solid foods (typically months 5–6 postpartum) [14, 15]. The costs for midwifery services, for both healthy women and those with complex conditions, are covered by health insurance (statutory and private) [16].

All women are free to choose their own midwife; no referral from family doctor or obstetrician is necessary. The women themselves must look for and contact a midwife, who may offer her services in the form of home visits or in her practice. By law midwives are entitled to offer antenatal care for the standard 10 to 12 statutory antenatal check-ups. Traditionally, however, women make an appointment with an obstetrician (not their GP) when they suspect they are pregnant and avail themselves of prenatal (medical) care, including at least three ultrasound scans. Some women opt for exclusive midwifery care during pregnancy, especially if they want to give birth at home or in a midwife-led freestanding birth centre (both options account for about 2% of all births) [17]. Some women prefer a model of shared prenatal care by both midwife and obstetrician.

In Germany midwives have the prerogative of providing birth support, meaning that a midwife can assist a woman to birth without a doctor present, but a doctor must call for a midwife when encountering a woman in labour. Only in case of complications (and most hospital births) is the midwife required to call a doctor (obstetrician/paediatrician) and to follow her/his instructions.

After discharge from hospital (typically at two or three days postpartum, but sometimes just a few hours after birth), mothers who have arranged postpartum care with a midwife can have a maximum of 36 home visits and phone calls for up to 12 weeks. In case of difficulties, such as preterm birth, and/or lactation problems, obstetricians or paediatricians can prescribe further midwifery support. In addition, midwives can support mothers when introducing solid foods and weaning. All costs are covered by health insurance, which is mandatory in Germany.

Nationwide, the three-year education of midwives was at secondary level (vocational training) until the year 2010, when academic study programmes were introduced by way of a model clause in midwifery law. Since then about a dozen four-year study programs for a Bachelor degree in midwifery have been established at colleges and universities (tertiary level). After passing the state exams, the graduates obtain permission to work as a midwife in any setting of the healthcare system. There is no regulatory body or compulsory registration of midwives; only those intending to practice as freelance/caseload midwives must notify the local health authorities of their intended practice.

### Array of midwifery services in Germany

Many women get to know a midwife when they visit a birth preparation class during the second trimester of pregnancy. These courses, which vary in content and according to the midwife’s preferences and practice focus, are typically offered for a total of 14 h either as a continuous weekly course or over a weekend. They are often for women and their partners, or some just offer a certain number of hours for partners; others are for single women. Professional care by midwives comprises preventive, supportive and monitoring services in terms of counselling, practical guidance and coaching, help in case of pregnancy complaints, and emotional support. Midwives promote the wellbeing of mothers and infants, foster processes of adaptation, ameliorate complaints, identify pathological deviations and refer to medical professionals. Furthermore, midwives are entitled to offer complimentary services, such as alternative treatments (e.g. acupuncture, homeopathy), and special emotional support for bonding or grief counselling, provided they have the required training and qualifications. Such complimentary services, some of which have to be paid for privately, are offered both by caseload midwives (47.6%) and those who are both employed in a hospital and self-employed (34.7%) [18].

### Interprofessional cooperation

According to the maternity care directive in Germany (the “Mutterschaftsrichtlinien”) [14], the findings of the preventive antenatal checks must be recorded in the pregnancy record book (“Mutterpass”) by either obstetrician or midwife. As the woman herself carries the record book with her, the course of the pregnancy is more or less apparent to the health professionals involved, depending on the quality of documentation.

Labouring women admitted to the maternity unit are cared for by midwives and obstetricians who are bound to follow the standards set by that hospital. Typically, the women do not know the attending midwife, unless they have met her during an antenatal check-up or tour of the delivery suite. Two hours after birth the women are transferred to the postnatal ward, where they are normally cared for by nurses; only some hospitals employ midwives there too. Caseload midwives (“Beleghebammen”), who have a contract with a particular hospital, continuously care for their labouring women (without or with consultation of an obstetrician), sometimes also on the postnatal ward, then for up to 8 weeks postpartum at home.

Vulnerable women and families living in very complex and psychosocially demanding situations (e.g. illicit drug use, domestic violence, minimal income/debt, social isolation), can be cared for by “family midwives”. They are specially qualified to assess the family’s special needs (together with the regional coordinator for early prevention of child abuse/a social worker) and support pregnant women and mothers until the first birthday of their child. They are either employed with the child protection services (regulated by social law), or are self-employed with a contract with municipal authorities. Their main focus is health promotion and “early prevention” by means of case management and interprofessional cooperation within and between the healthcare system and social services.

### Women’s experiences of midwifery care in European studies

**Fig. 1:**
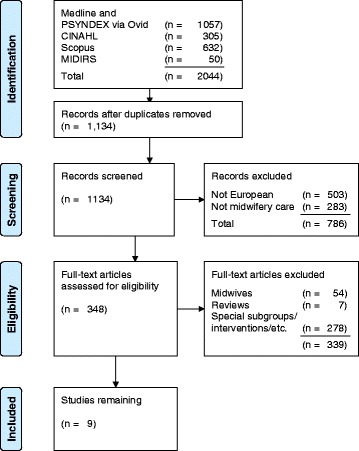
Flow-chart of identification and inclusion of relevant studies

In order to position the qualitative study presented here in a European context, and to provide adequate background on research done in European countries focusing on subjective perspectives of women on maternity care, a literature search was performed in the databases of MEDLINE, PSYNDEX, CINAHL, Scopus and MIDIRS. Criteria for inclusion were: qualitative studies in English or German using interviews, focus groups, or questionnaires with open-ended questions. The search was limited to research done in European countries (geographic) due to the heterogeneity of healthcare systems. Moreover, only literature published from the year 2000 onwards was included as laws and regulations pertaining to health care systems change over time (as has been the case in Germany). The terms of the search carried out in 2015 in the planning stage of the study and lastly updated in August 2016 were “perception”, “preference”, “need”, “understanding”, “perspective”, “experience”, “suggestion” or “subjective” AND “women”, “mother”, “midwifery” or “pregnancy”. From a total of 1134 results, 9 studies remained after exclusion of non-European studies (*n* = 503), studies not addressing midwifery care (*n* = 283), studies on midwives’ perspectives (*n* = 54), reviews (*n* = 7), and studies focussing on special subgroups or interventions (*n* = 278; see Fig. 1).

The results of the 9 eligible European studies are summarised below according to their insights into women’s subjective experiences in three areas: (a) women’s expectations of midwifery care, (b) choice of a midwifery care model, and (c) criteria indicating good midwifery care.

#### Women’s expectations of midwifery care

In four studies women reported on their expectations of midwifery care and care provision by the health system. Women wished for more continuity of care over the course of pregnancy, birth and the postpartum period. At any time in their care they wanted their midwife to inform and counsel them without any time pressure, and to focus more explicitly on their particular needs and interests [19]. In antenatal care women wished for appointments with their midwife in a calm atmosphere, conducive for building a relationship of trust and broaching sensitive issues, such as their experience of previous pregnancies and births. The women expected a humane monitoring of their pregnancy, which allowed for a holistic stance and sensitivity for the individual person [20]. Women not only benefited from reliable information, but eventually also – facilitated by the reassuring care of midwives – in terms of an increased self-confidence and equanimity with regards to their birth and parenthood. They appreciated a listening and holistic approach by midwives, emotional confirmation, pedagogical creativity, facilitation of new social contacts, and promotion of partner involvement [21]. Women called for meaningful antenatal classes which could also be attended by multiparae, and the option of getting to know the maternity unit and the midwives working there before onset of labour [22].

During labour and birth they wished to be provided with ongoing feedback and explanations pertaining to their progress. Regarding discharge, multiparae preferred to stay longer than the recommended three days and wanted more assistance during the postpartum period [22].

#### Choice of a midwifery care model

Regarding the issue of choice, women wanted to be in charge of their care and expected to experience pregnancy and birth as positive and important time periods of their life. They considered their own wellbeing secondary to the health of their child. They were willing to accept traditional, mostly biomedical maternal care, with the aim of safety and immediate expert help. In the women’s experience, the general practitioner (GP) acted as gatekeeper early on in pregnancy, actually deciding on the particular model of maternity care, typically steering the woman towards hospital-associated antenatal care, or a hospital birth. In some cases women had to resign themselves to hospital care due to the midwife’s requirements that they reach 38 weeks of gestation [23].

#### Criteria indicating good midwifery care

Four studies provided insights pertaining to quality of midwifery care. On critically assessing midwifery care, women reported that they felt left alone and unsupported during latent and early stages of labour, at home and in hospital [24]. They were intimidated by the noisy and unwelcoming hospital environment and the prevailing busyness and were concerned that they would receive unnecessary interventions if their birth didn’t progress as fast as expected [24]. The mothers critiqued the lack of personnel and information, which resulted in their feeling anxious, alone and unsupported, in a sense of loss of control, disempowerment, and ultimately a negative attitude towards a subsequent pregnancy. On the other hand, the women felt the midwives would have, or had, a major role in empowering them and enabling positive experiences [24].

Women who opted for a home birth, despite significant concerns and anxiety about potential emotional trauma [25, 26] after negative experiences associated with their previous hospital birth, felt emotionally supported and enabled to maintain control and dignity by their midwife. They gave birth at home feeling self-assured and content to have coped with labour without medical interventions [25].

Though women found it difficult, they participated in decision-making with their midwife in various birth settings and appreciated her/his attitude and active support [25, 26]. They experienced birth as a common venture, in which they and their partners were involved together with the midwife and other staff [26], and which deeply strengthened their self-confidence and self-assurance [25]. Women wished that the midwife promote individuality and adapt to their changing needs during labour and birth. They wanted to be supported in the uncertainty of childbirth, especially during transition times of labour. In their experience, the emotional and physical presence of the midwife and her woman-centeredness were not conceivable as separate from her “knowledgeable doing” [27].

They also stressed that it was important for them to be physically and mentally prepared for birth, to have information and guidance regarding the birth process and possible risks [23, 24]. This knowledge contributed to their feeling of safety, as did trusting relationships and a positive atmosphere in the maternity unit [26].

## Methods

The complete research project aimed at identifying the remembered experiences, needs and wishes of pregnant women and mothers in Germany which were of high priority for them regarding midwifery care. Thus, in an open and broad methodical approach (no pre-specified issues), empirical data were to be generated which could in future serve as a starting point for the development of a woman-oriented national agenda for midwifery research in Germany. The complete project encompassed focus groups of women (as users of midwifery care) and midwives (providers of midwifery care). As a large amount of data was collected and analysed, the focus here will be on *systemic aspects* of midwifery care as raised by pregnant women and mothers. S*ystemic aspects* entail modalities of midwifery care within the German healthcare system, such as access to, availability and model of midwifery care, choices offered based on national laws and regulations, and midwifery services as part of interprofessional maternity care.

### Aim and research question

The aim of the component of the study presented here was to identify the experiences, needs and wishes of pregnant women and mothers in terms of *systemic aspects* of midwifery care in Germany. The research questions were: a) what experiences and wishes do women in Germany have regarding midwifery and maternity care? b) What kinds of deficiencies and discrepancies do women experience when availing themselves of midwifery care in Germany?

Due to the limited scope of article publication, results of the study regarding other aspects of midwifery care, the four focus group interviews with midwives, and the determination of dominant themes for midwifery research will be published in a subsequent article.

### Design

This qualitative explorative research project was designed according to Gadamer’s hermeneutic approach [28], which aims at a broader, deepened understanding of the perspectives of other individuals. This was realised by an open approach, the focus on the other as subject of instruction, reflective dialogue amongst the research team, and explication of each step in data collection and analysis [29]. Firstly, the reflective dialogue centred on each team member’s own views on midwifery care for pregnant women, women in labour and birth, and postpartum mothers in Germany. This provided a “mirror”, and thus awareness of individual preconceptions, and allowed for clarified reflections and interpretation of participants’ experiences. Secondly, reflection and openness were called for during the focus group interviews: the women were allowed to relate their views without interruption by the researcher, and to converse amongst themselves without restrictions. Thirdly, a broad and context-related understanding of participants’ experiences, attitudes and values was aimed for by a thorough iterative process in data analysis (see below).

### Access to and sampling of participants

In this study “users” [1, 2] were pregnant women and mothers who had given birth in Germany within the past year (inclusion criteria); no other criteria were applied as participants with a wide variety of characteristics were intentionally sought. Women were to be excluded only if they were not sufficiently able to speak and understand German.

Pregnant women and mothers were informed about the study and invited to participate by multiple means: by case-load or hospital-employed midwives, who were contacted by email; via social media; and via the project website. The midwives were asked in particular to invite and encourage women with a low level of education. The sample was to consist of about 50% women whose highest level of education was equivalent to a Certificate of Secondary Education. Homogeneous focus groups of 4 to 6 people (either lower or higher educational level; and either pregnant women or mothers) were then formed, so that women wouldn’t feel intimidated about speaking up and relating their experiences and views, and in the assumption that smaller groups would facilitate the flow of speech and discussion.

79 women from 19 cities or rural areas in Germany were interested in participating. They were asked for a limited amount of sociodemographic data and some details with regards to their use of maternity care in a documentation sheet (see description below). In general, the focus groups were scheduled whenever several interested women from the same geographical area possessed the required characteristics for a particular group. However, for organisational reasons this was not always possible, and in three cases pregnant women and mothers were together in one focus group. The foremost aim of sampling was to include women with varied characteristics and to form focus groups which were heterogeneous with regard to age, parity, model of maternity care in pregnancy, and subjective rating of satisfaction with midwifery care.

### The sample

In total, 10 focus groups took place with a total of 50 women from 5 German federal states. Nine women had not attained an educational certificate or had less than an equivalent to a Certificate of Secondary Education, 8 women had an equivalent to a Certificate of Secondary Education, and 30 women had attained a higher educational level (see Table 1). Fifteen women were pregnant and 35 women had given birth during the past 12 months. All in all, 44 women had had one or more previous pregnancies. Several focus groups included women who had had a miscarriage or lost a child (*n* = 15). Six of these women were attending a peer support group for parents after stillbirth/infant death and wanted to specifically address their experiences and needs pertaining to the loss of their child; they were invited to a separate focus group. 16 women reported that their last pregnancy was “high risk”; 13 women were single mothers; and 4 women challenged by learning disabilities and living in a mother-child home formed a separate focus group.

**Table 1:**
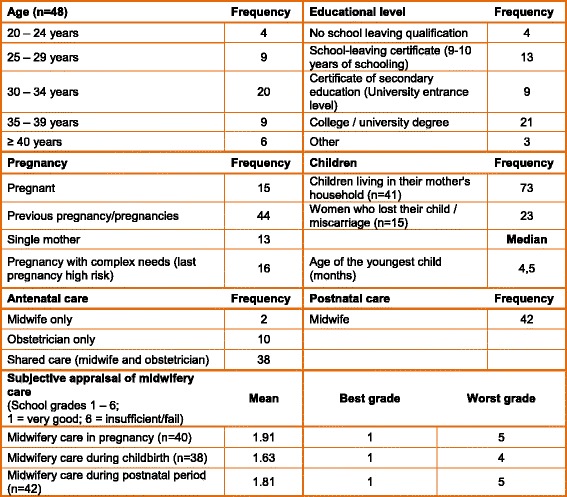
Characteristics of the participants (*n* = 50, if not otherwise indicated)

Six women were (additionally) cared for by family midwives due to their vulnerability with regards to limited resources and complex psychosocial needs. Two women received antenatal care solely from a midwife, 10 solely from an obstetrician, and the majority (*n* = 38) availed themselves of shared antenatal care (midwife and obstetrician). Participants appraised the midwifery care they had received by awarding the grades typically used in German schools, ranging from 1 (“very good”) to 6 (“insufficient/fail”). On average, the women in the sample awarded the grade 2 (“good”).

### Data collection

The focus groups were held over five months in 2015. The facilitators of the focus group interviews were two academically educated midwives who were members of the research team (EM and SL). The women were welcomed and the aim of the research project and participation in the focus group were (again) explained. The women chose pseudonyms to be used during the focus group interview. To begin with, a number of stimulus questions were offered by the facilitator, such as: "What was your experience of midwifery care?", "How did you want to be cared for in pregnancy/as a mother with your baby?", or "How should maternity/midwifery care be different?".

During the conversation, the facilitators mostly listened and did not interrupt. However, if the flow of conversation was stilted, as was more often the case with women of a lower level of education, the facilitator asked for more details or elaboration on the context to allow for deepened understanding. The focus groups generally lasted about 2 h.

All focus group interviews were digitally recorded and transcribed in full. The participants were protected by pseudonymisation of identifying names and specifics. Interested women who weren’t able to participate in the focus groups were offered the option of communicating their experiences and wishes regarding midwifery care in writing. Three women took advantage of this; their contribution was added to the total text material.

### Research team

Prior to the first focus group, the research team members documented their own experiences as midwives and mothers, as well as their knowledge about views of pregnant women and mothers known to them. As a team they reflected both on their corresponding and differing understanding of strengths and limitations of midwifery care in Germany, and on women’s experiences, needs and wishes from their own (midwife/researcher) perspective. During analysis differing views of team members became apparent in the incompatible understanding of text passages, necessitating deeper, more extensive reflection in order to grasp the essence of the participants’ perspective.

### Ethical aspects

The ethics committee of the Martin Luther University’s (MLU) medical faculty endorsed the arrangements being planned for the protection of participants’ rights. The participants gave their written consent after having been provided with ample information on the research procedure, pseudonymisation, voluntariness, and the possibility of opting out prior to the beginning of analysis.

The women were insured for the time of their travel to and participation in the focus groups, and compensated for their efforts with a gratuity of 70 Euro each at the venue.

The data file containing the names and contact data of interested and participating women was managed by a single team member (EM). This file and all other digital files (transcripts, analyses) were secured via a complex password. Final versions have been saved on the server (network computer) of the IGPW where they will be kept for 10 years.

### Data analysis

Focus group interviews and data analysis were performed parallel after four focus groups had taken place and been analysed. The focus group interviews were transcribed by a team member (ÄK) and the facilitator of each focus group then listened to the digital audio file to double check the transcript. The most relevant passages were found to be those in which women related their experiences of midwifery care emotionally, had very lively discussions about certain aspects, or expanded on them in more detail. The intention was to condense participants’ statements without losing important contextual detail of the related experiences.

**Fig. 2:**
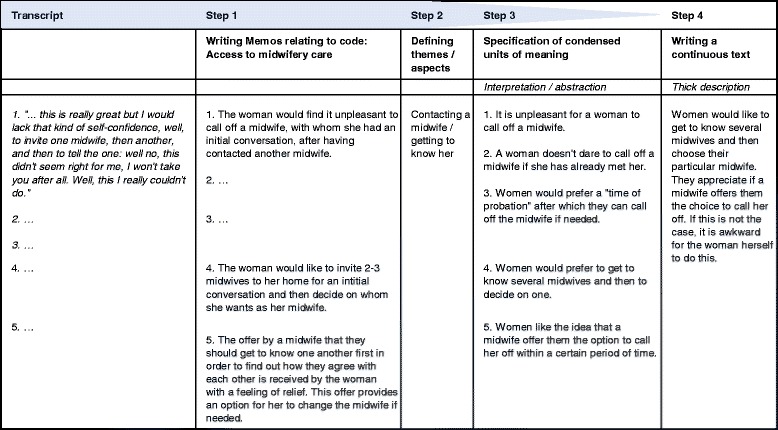
Depiction of the process of analysis regarding thematic subject “Access to and availability of midwives”

In accordance with Gadamer’s hermeneutic approach [28], the analysis was performed in a cyclical manner involving the following practical steps (see Fig. 2):

- Step 1: Two team members (EM, SL) independently analysed the transcribed focus group conversations using the MAXQDA software [30] by explicating the meaning of all relevant passages in “memos”. The independently recorded memos were then checked by a third team member (GA) regarding their content’s concordance. Memos which differed in their meaning (pertaining to the same text passage) were discussed until consensual understanding of the text passage was reached. Each memo was linked to a code (e.g. access to midwifery care), which served the purpose of putting the memos in structural order.
- Step 2: The memos of each code were grouped according to sub-themes (e.g. contacting a midwife/getting to know her).
- Step 3: The memos’ meaning (with related context) was extrapolated and abstracted. Thus “condensed units of meaning” were verbalised.
- Step 4: By way of discussion and all-day workshops the “condensed units of meaning” were repeatedly reflected upon with regard to their inherent dynamism, context and inter-relationships. They were gathered in common thematic subjects (e.g. “access to and availability of midwives”) and sub-themes (e.g. “selection of a midwife”). In order to fully describe women’s needs and wishes with regard to midwifery care the researchers (EM, SL, GA), wrote continuous texts (“thick descriptions”) which were checked for consistency with the previously documented “condensed units of meaning” by ÄK. Only a few smaller aspects were found to be unclear or missing, and were subsequently refined or incorporated.

The measures of quality assurance undertaken to strengthen the validity of the results were: independent steps of analysis, discernment processes in the team and numerous double checks. In the following results section study participants are not cited for two reasons: in contrast to individual interviews the flow of speech in a focus group is interactive and more fragmentary, and citation of longer passages is beyond the scope of this publication.

## Results

Three common thematic subjects entailing women’s experiences and wishes and their perceived deficiencies and discrepancies regarding *systemic aspects* of midwifery care in Germany (see Table 2) resulted from the analysis. They are:

- Knowledge or lack of awareness of midwifery care (4 sub-themes)
- Access to and availability of midwives (5 sub-themes)
- Midwifery care in the healthcare system (6 sub-themes).

**Table 2:**
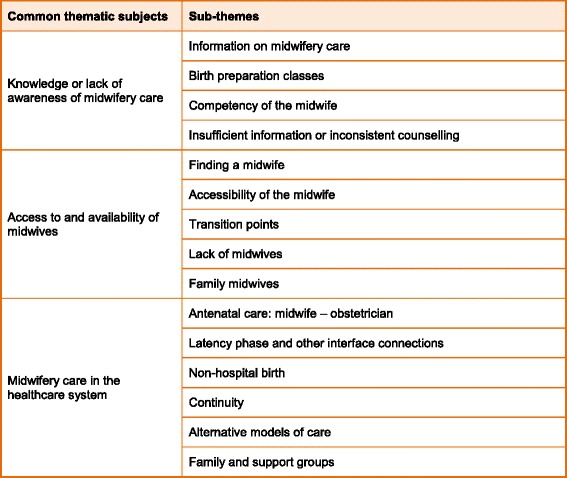
Common thematic subjects and their sub-themes

In the following descriptions no indication of “frequency” (e.g. “some” women) will be given and the general term “women” is used, as the inherent focus is not on individuals, but on conceptual content in relation to a respective context.

### Knowledge or lack of awareness of midwifery care

#### Information on midwifery care

It’s often by chance that women receive information on midwifery care, as they have no (easy) access to relevant information. Women want more information about the scope of midwifery care and the regulatory framework and services of the healthcare system as they are unsure which services are covered by health insurance. They are unaware that midwives offer antenatal care even to those pregnant women diagnosed with risk factors.

Although women prefer information given orally, they also appreciate brochures, or a note in the pregnancy record book (“Mutterpass”) advising on the scope of midwifery services. Women complain that the pregnancy record book is not really designed for their own convenience: they miss user-oriented checklists for pregnancy, birth, and the postnatal period, and a layout which would facilitate antenatal care which is shared by obstetrician and midwife. They criticise and feel anxious about the fact that they cannot understand the medical findings documented in it and what their meaning is with regard to their baby’s development or necessary medical interventions.

Women expect and wish that their obstetrician inform them very early in pregnancy about midwifery care: that it is up to them to look for a midwife themselves and make contact early on, that midwives offer support in pregnancy in addition to antenatal courses, and the difference in the scope of care offered by midwives and family midwives.

Women are unaware of the various kinds and concepts of birth preparation classes and postnatal exercise courses (both of which are also offered by midwives and covered by health insurance in Germany). They are also usually unaware of the option of midwifery support for introduction of solids and weaning.

In hindsight, first-time mothers and women who have had a miscarriage felt they profited, or would have profited, from midwifery care in pregnancy.

#### Birth preparation classes

In women’s view the various antenatal classes should be standardised so that every woman receives a minimum of information and exercises, independent of the particular class she attends. They want detailed information in advance on the philosophical stance of the midwife holding the class and on the content of each course hour. In case of inability to attend they would appreciate an extra course hour to make up for content missed. Reasons for non-attendance of antenatal classes are: lack of knowledge and appreciation of the course content, no interest in meeting other pregnant women, course location geographically too distant, or perception of being able to manage without.

Women expect the full scope of information to be presented in antenatal classes, especially on physiological birth. They hope for exercises which are body oriented, and/or foster breathing and vocalisation; tips for relaxation, wellbeing, coping with labour pain, and attachment/bonding with their child.

Women appreciate special course hours for their partners. In their view, the courses offered at hospitals should also address other potential birth settings, such as birth centres or homebirth. They also want to be prepared for parenthood and the time after birth.

#### Competency of the midwife

As women seem to lack knowledge regarding midwives’ scope of practice, they only have a limited idea of the potential benefit of their expert support. For example, women are unsure about whether midwives need additional training for non-hospital births. They are frequently unaware of their competency to provide antenatal care, perform vaginal exams, check the perineum and stiches, and perform the newborn screen.

As they trust her competency to decide when a woman should go to the clinic, women would prefer a midwife to do check-ups when they have passed the expected date of birth, and counsel and support them during latency phase.

In the antenatal and postnatal wards in hospital, women often cannot recognise who is a midwife and who is not. Shortcomings they experience are thus ascribed to midwives, even when there are no midwives on staff.

#### Insufficient information or inconsistent counselling

Multiparae complain that they are not given the same level of information, counselling, and midwifery support as in a first pregnancy. On the whole, women differ in their expectations of midwifery care: some rely on the proactive support of midwives, some expect support-on-demand by midwives (tendency to overestimation), and others believe that they don’t need any support (tendency to underestimation).

Apart from women’s experiences that support measures and counselling were either positive or negative, they often felt that the counselling they received from midwives came too late.

Women feel distressed by differing diagnoses and inconsistent counselling by doctors or midwives, such as those pertaining to medical interventions, SIDS prophylaxis and kangaroo care, procedures in cases of post-term pregnancy, or breastfeeding recommendations. The differing views of experts confuse and stress women, especially first-time mothers, as they don’t constitute options or choices per se.

Those women who have the means to do so search for additional information and alternative options in books or online (including apps), where they typically find differing pieces of information. They wish and expect doctors or midwives to counsel and help them filter information according to their individual situation and needs. Women appreciate midwives explaining to them which interventions are evidence-based and which are overused. This reduces the emotional pressure they experience regarding, for example, medical antenatal screening options. They expect to be provided with the rationale for interventions and also for non-intervention.

Women with limited education or limited communication capacity need special attention from midwives and possibly repeated explanations of processes, procedures, and interventions. In order to facilitate such women’s access to midwifery care and foster their mothercrafting, an interprofessional network is needed (in addition to effective collaboration between midwife and obstetrician), in which pregnancy counsellors, social workers, paediatricians, and early child development experts work closely together. Midwifery care in the postpartum period should not be concluded early in such cases, as a longer process of educational coaching is needed. This role can be filled in Germany by family midwives, who accompany women with limited health literacy, communication resources, or mental capacity until their child’s first birthday.

### Access to and availability of midwives

#### Finding a midwife

Women find the search for a midwife frustrating and stressful due to the large number of calls necessary and the subsequent refusals. In particular those with psychosocial problems, with limited literacy, and women with difficulties in pregnancy are overwhelmed by the task of looking for a midwife. Often they start too late and as a result are unable to obtain antenatal midwifery care.

Multiparae, who are already known by their midwife, find it easier to book midwifery care and to be accepted for an antenatal or postpartum course. Women in full-time employment, who have recently moved, and those without internet access find it particularly difficult to find a midwife.

Women want information well in advance on the various services offered by individual midwives, especially whether they provide antenatal care or not. Women suggest an internet platform with various functions (e.g. search by postal codes), including photos, information on the midwives’ philosophical stance, their values, their positions regarding midwifery care, and whether they still have availabilities.

Women would like to get to know several midwives and then choose their particular midwife. They don’t want a midwife assigned to them by others. They appreciate being offered the chance to decline a midwife’s services, as it can be awkward for the woman to do this otherwise; they therefore usually rely on the recommendations of friends. However, they would prefer that midwives inform them about the services of colleagues and particularly about midwives who offer birthing care in other settings than themselves.

#### Accessibility of the midwife

Women appreciate the accessibility of the midwife “at all times”. They expect their midwife to clearly communicate when she can be reached and by which method, as a general rule, or in case of an emergency. Alternately, they would prefer a hotline staffed by competent personnel which they could call at any time without compunction. In their view, telephone counselling would spare them unnecessary visits to emergency rooms, overuse of interventions, not to mention the time and energy, and the inconvenience for them and their newborn.

Women criticise the following experiences which are judged as unreliability of the midwife: poor effort on the part of a caseload midwife to make contact with them, delayed scheduling of appointments, repeatedly cancelling home visits, no offer of alternative appointments when women need to cancel an appointment, and an unexpected end to care.

Women expect their midwife to organise a substitute midwife to cover for her during absences, and to inform her about the women’s needs and wishes.

#### Transition points

Women in the latency phase or during induction feel forsaken if they cannot stay in the obstetrical unit and are sent home or transferred to the ward, or if they are not (well) cared for by a midwife on the ward.

Postnatally, on discharge from hospital, women expect the midwife to visit on the same day, even on weekends and holidays. An early discharge may be advantageous for women exposed to inconsistent breastfeeding counselling by hospital staff. In such cases, their caseload midwife can support them from the beginning in their endeavour to breastfeed successfully.

#### Lack of midwives

Due to a lack of midwives, women feel compelled to look for a midwife early in pregnancy, i.e. the first trimester. The perceived or actual insufficient number of midwives has various reasons: not all midwives offer the full scope of midwifery services, some work only part-time, the philosophical stance of midwife and woman may be incompatible, and there are not enough midwives in some cities and rural areas. Especially in case of a miscarriage or preterm birth, it is difficult or even impossible for them to find a midwife at short notice.

Women understand that midwives are busy and care for a number of women at the same time. However, they are disappointed when midwives take on many cases, and in the end do not have sufficient time for individualised care. They contrast this observation with midwives who accept only a few cases justified by their limited capacity, who provide high quality care, and who organise an effective substitution if needed.

#### Family midwives

Women often do not know what the difference is in the scope of a ‘midwife’ and a ‘family midwife’. If they are aware of it, they appreciate the additional care by the family midwife which can be provided either parallel, consecutively, or by the same person. Women requiring additional support find it helpful when the family midwife accompanies them to medical appointments, explains medical terms, and provides support up to the first birthday of the child.

### Midwifery care in the healthcare system

#### Antenatal care: Midwife – Obstetrician

Women are aware of the differences in their respective approaches in care and wish they would be on an equal footing. In contrast to their expectation of an effective cooperation of obstetricians and midwives in antenatal care they experience conflicts, particularly when the obstetrician and the midwife do not appreciate each other’s contribution to care. Women who avail themselves of antenatal care by both professionals may be subjected to high emotional pressure: they feel they must defend their decision to also have midwifery care, or conceal it from the obstetrician.

Women expect that they and their partners be given comprehensive information by both professional groups (e.g. regarding breech presentation, use of medication, indications for antenatal CTG). With some complaints, such as back pain and pain in the lower extremities, they feel not really cared for and left to cope alone. Women expect doctors to refer them to midwifery care also when classed as ‘high-risk’ (e.g. gestational diabetes).

#### Latency phase and other interface connections

In latency phase women feel unwelcome in hospital and poorly cared for if they are found not to be in established labour and are sent to the ward or back home. During induction they are sent for a walk outside the labour unit, or they are given various medications and interventions based on justifications incomprehensible to the women. When they stay at home women are insecure and lack competent guidance and practical support. They would expect a midwife to visit them at home and stay with them, and that they decide together when to go to the hospital. In such cases they could spend longer at home and the midwife could inform her colleagues in the hospital about her findings.

In order to avoid unnecessary interventions, women would prefer midwifery care during pregnancy and birth, with the doctor only being consulted if desired. Women would be less stressed if the communication and cooperation amongst midwives and that between midwives and doctors were more effective, and the women themselves did not have to convey the findings of one to the other.

Especially after caesarean section or a stillbirth, women feel they are discharged from hospital much too early and are left to manage the interface by themselves without an effective hand over, or hand over protocol.

If their midwife cannot provide the help needed, women welcome referrals to other experts, e.g. for dietary or psychological counselling, or to out-patient clinics for infants’ regulatory problems (e.g. fussing, crying inconsolably). In particular in cases of infant death, midwives who are not trained in grief counselling should refer parents to an expert and to a support group and support them in making contact with them. Nevertheless, in their view, care by other professionals cannot replace midwifery care.

#### Non-hospital birth

Women expect to be able to give birth in non-hospital settings, such as a birth centre, also in the future. For them, the birth centre is an optimal setting for childbirth: they appreciate the atmosphere, the continuous care by their midwife, the support for a physiological birth process, and the absence of medical interventions. However, presently they feel they must be very courageous to stand up for their decision for a non-hospital birth. Another attractive option for them would be an “outpatient birth” with discharge from hospital within 24 h postpartum.

#### Continuity

Women would want continuous care by their chosen midwife from conception until weaning. In the women’s view, continuous care by a midwife, or a team of midwives, is best realised in a birth centre, or in case of a home birth. In their view continuity would provide them with ample support to achieve a normal birth and to avoid unnecessary interventions. Medical interventions should be indicated only in case of complications.

Due to the fact that maternity care in Germany is fragmented on the whole, women are faced with the dilemma that while they are able to choose a midwife for antenatal and postpartum care, in hospital they are assigned an unfamiliar midwife. Single mothers in particular expect continuous care by a midwife as they are more likely to feel left alone. Even women having a caesarean section want to be continuously cared for by a midwife.

#### Alternative models of care

In the future, women would want their midwives to be allowed to perform the ultrasound scans so that antenatal care can be solely managed by midwives. Women prefer not to make use of additional tests which they have to pay for out of pocket. Pregnant women and mothers would like consultation hours to be offered by a midwife in a family centre.

Disadvantaged women with limited formal schooling who cannot recall detailed information would find it helpful if their midwife would accompany them to doctors’ appointments in pregnancy and after birth. This would be of benefit for them and for their infant’s health and mothercrafting. In cases of learning disability they also call for alternative courses which take their special needs into consideration. Alternate antenatal and postpartum courses for pelvic and abdominal muscle training could be re-conceptualised as classes and combined with home visits, ideally extended over a prolonged period.

#### Family and support groups

Women expect that midwives more explicitly facilitate the involvement of their partners and family members in their care, starting in hospital and continuing at home. This would tap into available resources and relieve them in caring for the infant. In hospital women want to be offered a family room and encouragement to articulate their experience of birth.

Birth preparation classes, the most common support groups, are attended by women in order to get to know other pregnant women, to familiarise themselves with the birth unit at the hospital, to consider issues pertaining to birth and the postpartum, and to devote time to consideration of their unborn child and their pregnancy. Psychosocially vulnerable women however may dread being with peers with whom they cannot form, or don’t want, longer lasting relationships.

Apart from antenatal classes, women are calling for peer groups which provide support and affirmation with regard to negative birth experiences as well as information about other health professionals, alternative stances and remedies, and activities in self-care or infant care unknown to them. Ideally, they should be accompanied by an expert, such as a midwife.

## Discussion

### Discussion of methodical aspects

The range of methods of inviting interested women in different geographical regions of Germany to participate in focus groups provided for a selection of participants with a variety of sociodemographic characteristics. Even though the aim of four focus groups with women who hadn’t acquired an educational level equivalent to a Certificate of Secondary Education could not be achieved, a substantial number of women with a low level of education were included. In addition, women with complex health and psychosocial needs and support requirements participated in the focus group interviews. The experiences of women living in a mother-child home and women who had lost their child gave valuable insights into their particular needs and wishes with regard to midwifery care. On the other hand, due to the requirement of participants needing to be able to communicate well in German, the viewpoints of immigrant women are most likely insufficiently represented.

In the focus groups with women who had a similarly high level of education, the conversations seemed rather uninhibited and free flowing as the women were eager to communicate their experiences, needs and wishes. This made it easier for the researchers to grasp narrative, meaning, and strands of argumentation. In contrast, analysis of the conversations of women with lower levels of education, or those who did not complete formal schooling, proved to be more challenging: The women didn’t seem at ease engaging in free-flowing conversation and speaking in more detail on a particular subject. Although the facilitators stimulated the conversation with intermittent open questions, the women often highlighted certain experiences or attitudes without providing the context. In this regard, it was hard to adequately grasp the participants’ viewpoints at various times, making it difficult to be sure the meaning was fully comprehended.

On the whole, the iterative interpretative hermeneutic analysis of the data according to Gadamer [29] was a meaningful and practical method for exploring the participants’ views on midwifery care in Germany. The team of four researchers proved to be an asset in assuring validity and reliability of the data and results. It allowed for independent steps in the analysis and repeated double-checking of data and interpretation. With regard to generalisation of the results, the heterogeneous sample of women provided for a broad and varied spectrum of experiences regarding midwifery care in Germany. Still, had additional focus groups taken place, other views might have been encountered. With regard to the analytic process it must be contended that – despite definitive analytical steps, double checking, and prior reflection and documentation of the teams’ subjective assumptions – other researchers might have abstracted and condensed the data in a different manner, based on their particular professional experience and mindset. Despite the limitations mentioned, the results are deemed valid (grounded in data), reliable and meaningful for informing midwives in Germany and abroad about women’s needs and wishes regarding midwifery care throughout the perinatal period.

### Discussion of results

#### Knowledge or lack of awareness of midwifery care

A major criticism regarding the arrangement of midwifery care within the German healthcare system addresses the lack of easy access to relevant information, especially about the services and scope of practice of midwives. The women criticise that obstetricians do not refer them to midwives (early enough) for antenatal care, support and counselling as is indicated by social law §24 SGB V [13]; their recommendation merely focusses on the attendance of a birth preparation class.

Moreover, women are not well informed about the models of care, the necessity and indication of interventions, evidence-based options of care, and choices they have. An evaluation of 13 meta-syntheses for a framework for quality maternal and newborn care (Lancet series) [31] confirms how important it is for women to have timely access to information and to gain an early understanding of the various options of maternity services within the healthcare system. This means that women need to be comprehensively informed about midwifery services, midwives’ competencies, evidence-based interventions and their own rights and choices as users of the healthcare system, not only during individual consultation, but also by means of freely available written statements or easily accessible online platforms. In particular, methods of making this information readily available to women who have limited formal schooling, learning disabilities, no online access, or insufficient German language abilities need to be explored.

#### Access to and availability of midwives

The frustration and stress experienced by women looking for, and trying to contact, a midwife was a dominant theme in the focus groups. When in the end they had actually contracted a midwife, they appreciated the midwife’s accessibility and dependability; in their perception, this is a criterion for good midwifery care.

Women’s disappointment when midwives had too little time for individual care due to case overloading corresponds to the perceptions of other European women [19, 20, 22]. They wished for woman-centred counselling with no time pressure, proactive provision of information, on-going feedback and explanations during labour and birth, and for development of a relationship of trust with their midwife. With regards to discharge from hospital, women expected more flexibility and regard for their individual needs [14], especially after a caesarean section or in the event of having lost of their child.

Women in Germany and other European countries appreciate various qualities of midwifery care: the midwives’ supportive and holistic approach [21], their proactive and preventive counselling, and a trusting relationship [20, 26, 27]. The midwife’s expert support fostered women’s self-confidence, strength, sense of control [21, 25, 26], participation in decision-making [26], and dignity [20]. Women in Germany, moreover, stress that support from a midwife is important early on in pregnancy so that a trusting relationship can develop.

#### Midwifery care in the healthcare system

In antenatal and postnatal care not only first-time mothers, but also multiparae wish for the full scope of counselling and midwifery support. They prefer to be given comprehensive information on preventive measures, tests, diagnoses and interventions [20]. Some participants were particularly critical of the fact that effective care in cases of severe pain of the lower back and/or extremities was not offered, by either midwife or obstetrician, leaving them to cope with significantly restricted movement which impacted on their ability to perform daily activities. There is no systemic pathway in maternity health care which prescribes a consultation of physiotherapists, or orthopaedic surgeons.

The women in this study called for a standardised minimum of information for birth preparation classes promoting prenatal attachment, body awareness, relaxation, and coping during physiological birth. As in the study by Hildingsson et al. [22], they deemed peer groups important, suggesting that they be mentored by a midwife who provides guidance and literature. In particular, women wished that they and their partners receive the same information during pregnancy, birth, and the postpartum period. This would enable their partners to more effectively support them in decision-making and to relieve them in caring for the infant.

A systemic deficiency in the provision of midwifery care becomes apparent in latency phase or during induction of labour as in the studies by Borrelli et al. [27] and Larkin et al. [24]: the women are not competently supported in either phase. At home, they cannot avail themselves of midwifery care unless they are planning a home birth. They feel insecure and left alone and therefore go to the obstetrical unit much too early when labour is not yet fully established. There they are not welcome and are usually sent to “walk around” or are admitted to the antenatal ward, where the women feel they don’t belong. This is a typical case of poor management of transition points; a systemic deficit. However, based on past judicial decisions in Germany, which determined that the latency phase is part of the birthing process, only midwives who pay high liability premiums for the provision of out-of-hospital care see themselves in a position to support women during that period. They number far too few to provide care for all women who give birth in hospital (98% of all childbearing women). The significance of support in the latency phase was underlined by women in Sweden [26], whose experience of continuous support and guidance from their midwife, beginning in the latency phase, contributed to positive memories of their birth. Besides the lack of expert support, women in early labour were concerned about receiving unnecessary medical interventions in hospitals in Germany, as in other countries [24, 27]. The Lancet quality framework [31] calls for initiatives in midwifery care during the latency phase so that women are only admitted when they are actually in active labour.

The women in the focus groups called for better options for continuity of care from the beginning of pregnancy until weaning [19, 32], either by a team of midwives or a midwife led unit. They criticised that they go to great trouble to find a midwife for antenatal care and pregnancy support, and when they go to hospital for their birth, the most special experience in their lives, they are cared for by midwives they do not know and with whom they had no chance to build a trusting relationship. This disruption of continuity of care, and the related development of alternative models of care, requires further exploration in the future [31].

With regards to interprofessional cooperation, the women in Germany were distraught when their midwife and obstetrician had conflicting approaches to care, especially regarding their choices for non-intervention or out-of-hospital birth. The participating women called for open documentation and communication between midwives and obstetricians ensuring an effective and cooperative maternity care in the interests of the women concerned.

The women’s openness for “consultation with and referral to other services” is mirrored in the definition of the practice of midwifery within the framework for quality maternal and newborn care by Renfrew et al. [31].

An important insight of this study, which has not been found in other European studies, refers to vulnerable women: they require special attention by midwives. Women with limited formal schooling and psychologically vulnerable women wished for antenatal and postnatal classes specifically tailored to their needs. This is of significance, as vulnerable women are more likely not to attend mainstream birth preparatory classes and postnatal group courses in which they feel out of place. Some also don’t see the need for midwifery care after a hospital birth. They therefore lack vital information on both physiological changes and pathological deviations in pregnancy, birth, and the postpartum period.

They called for more time in midwifery care for counselling and for reviewing and deepening the knowledge acquired. Moreover, they also appreciated the additional and extended support by family midwives up to the first birthday of the child. In the event of having lost their child in pregnancy or after birth, the women called for particularly sensitive communication, ample time for them and their partners to farewell their dead child, expert grief counselling, and liaison with peer support groups.

## Conclusion

Future development of midwifery care within the healthcare system in Germany must address a number of issues: one, the provision of, and easy access to, standardised information about midwifery scope of practice and services for women and parents, irrespective of their educational level; two, effective cooperation between midwives and obstetricians in the interests of the women they care for, beginning in early pregnancy, as well as the effective management of transition from one setting/professional to another; and three, quality assurance, particularly with respect to antenatal courses, support during latency phase, and intrapartum care in hospitals.

The various models of midwifery care options must be timely and effectively communicated to all pregnant women. New models must be developed in the following areas: booking management, to alleviate frustration and stress of women trying to contact a midwife; options for continuity of midwifery care from the beginning of pregnancy until weaning; forms of special midwifery care for vulnerable women which adequately address their individual needs in terms of information, practical support and coaching, liaising with support peer groups, and referral to other experts.

Women expect more from midwives than just medical care; they expect a holistic respectful approach which attends to their physical, emotional, and social needs, as well as their individuality in experience, viewpoints and behaviour. Moreover, they need the midwife as their advocate for physiological childbirth, proactively offering them evidence-informed counselling and practical support for coping in a self-affirming manner. Women’s partners and/or families need to be involved in information giving sessions and decision making as desired and appropriate, so that women receive the necessary social support, whether or not the midwife is present.

## Abbreviations

DFG: Deutsche Forschungsgemeinschaft
GP: general practitioner
IGPW: Institute for Health and Nursing Science
MLU: Martin Luther University
PEKiP: Prager parent-child programme (Prager Eltern Kind Programm)
SGB V: Sozialgesetzbuch V (social law) - Gesetzliche Krankenversicherung
SIDS: Sudden Infant Death Syndrome
